# MiRNA 106a-5p in cerebrospinal fluid as signature of early relapsing remitting multiple sclerosis: a cross sectional study

**DOI:** 10.3389/fimmu.2023.1226130

**Published:** 2023-08-30

**Authors:** Aurora Zanghì, Virginia Manuti, Gaetano Serviddio, Emanuele D’Amico, Carlo Avolio

**Affiliations:** Department of Medical and Surgical Sciences, University of Foggia, Foggia, Italy

**Keywords:** multiple sclerosis, cerebrospinal fluid (CSF), oligoclonal bands (OCBs), biomarkers, MicroRNAs, early disease biomarkers

## Abstract

**Background:**

Circulating microRNAs (MiRNAs) have been investigated for their role in fine-tuning the adaptive immune response to inflammatory factors and in Multiple Sclerosis (MS). They have been investigated as possible biomarkers for the diagnosis and prognosis of the disease.

**Methods:**

A cross sectional study conducted at the MS centre of Foggia, Italy. We enrolled patients with (1) an age between 18 and 55 years, (2) a definitive diagnosis of relapsing remitting MS (RRMS) as per the revised McDonald criteria, and (3) naïve to any disease modifying therapy (DMTs), as well as (4) patients with other neurological disorders (OND). The aim of the study was to compare the levels of expression of miRNA 21-5p, miRNA 106a-5p, miRNA 146a-5p, and miRNA223-3p in cell-free cerebrospinal fluid (CSF) in RRMS patients and OND. Investigated MiRNAs were extracted, retrotranscribed, and then assessed by real-time polymerase chain reaction assay (q-PCR). A receiver-operator characteristic (ROC) curve was used to test MiRNAs as a biomarker for diagnosing MS. A linear regression analysis was done to find any association with disease characteristics at the time of diagnosis.

**Results:**

A total cohort of 70 subjects (70% women) was analyzed. Out of them, 35 had a RRMS diagnosis. MiRNA 106a-5p (7.8 ± 3.8 vs 1.3 ± 0.9, p=0.03) had higher levels in RRMS patients when compared to OND. The ROC curve indicated that MiRNA 106a-5p could be considered as a disease biomarker with an area under the curve of 0.812 (p<.001; 95% CI 0.686-0.937). Linear regression analysis showed an association between the number of oligoclonal bands and MiRNA 106a-5p levels (B-coeff 2.6, p<.001; 95% CI 1.3-4.9).

**Conclusion:**

We described miRNA 106a-5p as a possible signature in the CSF of RRMS patients in early phases of the disease. Further studies are needed to characterize its role in early MS as a disease biomarker.

## Introduction

Multiple sclerosis (MS) is an inflammatory-mediated demyelinating disease of the human central nervous system (1, 2). The guiding principle of diagnosis is that of dissemination in time and dissemination in space and is based on clinical and radiological features on magnetic resonance imaging (MRI) (3–5). The research of biomarkers for early recognition of the disease has brought increased research on blood or cerebrospinal fluid (CSF) parameters as possible disease biomarkers (4–8). However, there is no single diagnostic laboratory test specific for MS (4). The disease may be promoted or influenced also by environmental elements or epigenetic mechanisms.

MicroRNAs (miRNAs), small non-coding single stranded RNA molecules, can regulate gene expression at the post-transcriptional level by binding to target messenger RNAs, leading to their degradation or translational repression (5, 9–11).

MiRNAs have been analysed in different tissues (peripheral blood mononuclear cells, CD4+ T cells, and MS brain lesions) but they can be isolated also in body fluids such as plasma or CSF (5, 12–17).

CSF is considered a reliable source of biomarkers because it can reflect the biochemical changes in the brain (5, 16, 18, 19). These studies led to the identification of a number of miRNAs as candidate MS biomarkers, such as MiRNA 21-5p, MiRNA 106a-5p, MiRNA 146-5p, and MiRNA 223-3p, suggesting that these miRNAs may be used as a signature for MS and could play critical roles in MS pathogenesis, although further confirmatory studies are needed (4, 5, 9, 12, 15).

This study aims to evaluate the levels of expression in cell-free CSF samples of MiRNA 21-5p, MiRNA 106a-5p, MiRNA 146-5p, and MiRNA 223-3p compared with individuals with other neurologic diseases (OND) and to study their association with disease characteristics of relapsing remitting MS (RRMS) diagnosis to assess their possible value as biomarkers of RRMS diagnosis.

## Methods

### Participants

This is a cross sectional study, conducted at the MS centre of Foggia, from 1 January 2022 to 31 December 2022. We enrolled patients with (1) an age between 18 and 55 years, (2) a definitive diagnosis of RRMS as per the revised McDonald criteria (3), and (3) naïve to any disease modifying therapy (DMTs), as well as (4) patients with OND. The list of diagnoses for the OND group is given in **Table e1**. Patients receiving other immunosuppressive/immunomodulant drugs for other diseases or exposed to steroids within 30 days of initial blood collection were excluded.

### Procedures

#### Clinical and neuropsychological assessment

All information on the clinical and radiological data of RRMS patients were retrospectively collected within 12 months from enrolment. The following data were collected: a) demographical (age, sex) b) clinical (disease duration, disability assessed by the Expanded Disability Status Scale (EDSS), and number of relapses in the year before diagnosis), and c) radiological (the number of brain and spinal cord magnetic resonance imaging (MRI) lesions on T2 weighted sequences and number of patients with brain MRI lesions on T1 gadolinium-weighted sequences). The MRI obtained within 6 months of a confirmed diagnosis was considered as the baseline MRI; all scans were obtained using the same 3-Tesla machine (Siemens, Magnetom Skyra).

Cognitive performance was assessed using the Symbol Digit Modalities Test (20). Z-scores were calculated accordingly to normative values.

All information was retrieved from an iMed^©^ software database (iMed^©^, Merck Serono SA, Geneva).

### CSF collection

Lumbar puncture was routinely performed as part of the diagnostic workflow. Informed consent was provided for the procedure. The CSF parameters included were white blood count, total protein and CSF/serum albumin ratio.

IgG oligoclonal bands (OCBs) were quantified in serum and CSF using an Immage 800 nephelometer (Beckman Culter, Nyon, Switzerland). They were analyzed and quantified by isoelectric focusing and immunoblotting. Link index was also collected (CSF IgG to CSF albumin to the ratio of serum IgG to serum albumin) (20). This ratio-of-a-ratio, when greater than 0.7 (or the defined value for the laboratory), was indicative of intrathecal synthesis of IgG (21).

Cell-free CSF was stored at -80°C until miRNA extraction. It was obtained after immediate centrifugation after its collection at 1,000xg for 10 min at room temperature.

### Analysis of circulating miRNAs

#### Circulating miRNAs extraction and purification

CSF was thawed at room temperature. miRNA was extracted from CSF using the miRNeasy Serum/Plasma Kit according to the manufacturer’s instructions. The extraction was performed starting from a volume of 200 μL and mixed with 5 volumes of QIAzol Lysis Reagent. Then, 200 μl of chloroform was added. After centrifugation (12,000xg, 15 min, 15°C), the upper aqueous phase was recovered and mixed with 1.5 volumes of 100% ethanol. After being placed into a RNeasy MinElute spin column, three washing steps were performed.

Total miRNA was eluted with 14 μL of nuclease-free water. The absorbance was measured at 260 nm with a NanoDrop 1000 Spectrophotometer.

#### Circulating miRNAs Reverse Transcription

miRNAs were reverse transcribed using a miRCURY LNA RT kit and we used a fixed volume of 1.12 μL of miRNA for the reverse transcription (RT) to reach a final volume of 6.5 μL with nuclease-free water according to the manufacture’s instruction.

### Analysis of individual miRNAs

The resulting cDNA transcript was used for polymerase chain reaction (PCR) amplification using miRCURY LNA SYBR Green PCR Kits and a miRNA specific prime set (miRCURY LNA miRNA PCR Assay). Individual gene expression was assessed by RT-PCR using the Applied Biosystem 7300 Real-time PCR System and duplicates for each sample were performed. Expression values in CSF were normalized using the miRNA-16 5p reference gene as endogenous control. Relative expression of miRNA 21-5p, miRNA 106a-5p, miRNA 146a-5p, and miRNA 223-3p was analyzed using the comparative Ct method (2^^-ΔΔCT^) miRNAs panel, and endogenous controls were chosen according to the literature in the field and previously published studies from our research team (22, 23).

### Statistical analyses

Categorical variables are presented with counts and proportions, while continuous ones as the mean ± standard error of the mean (SEM) or median with interquartile range (IQR). Data distribution was assessed using the Kolmogorov–Smirnov test. The Mann–Whitney test was used to compare variables of interest.

Receiver operating characteristic (ROC) curve analysis was used to determine the power (sensitivity and specificity) of miRNAs indices as discriminatory biomarkers for the diagnosis of MS. The area under the curve (AUC) was also evaluated.

A linear regression model was built to test any statistically significant associations between miRNAs and CSF characteristics (OCBs number, Link index) and disease characteristics (baseline EDSS, number of relapses in the year before diagnosis, number of patients with lesions on T1 gadolinium-weighted sequence on baseline MRI, SDMT z score). B-coefficients, 95% confidence interval (CI), and p-values were reported. A value of.05 was considered significant. Using Cochran’s formula, the sample size was calculated. SPSS statistical software version 21.0 (IBM, Armonk, NY) was used for all analyses.

## Results

A cross-sectional study of 70 subjects (70% women), with a median age of 33 (IQR 28-47) years, was analyzed. Out of them, 35 had a RRMS diagnosis. All RRMS patients were naïve to any DMT and had a short disease duration (4.2 ± 1.6 months). The mean number of relapses in the 12 months before diagnosis was 1.6 ± 0.5. Gadolinium-positive lesions were present in 62% of the patients. Demographical, clinical, radiological and CSF characteristics are shown in **Table 1**.

**Table 1:** Demographical, clinical, radiological and CSF characteristics.

	Whole cohort (n=70)	OND (n=35)	RRMS (n=35)	p-value
Female, n (%)	49 (70)	25 (71.4)	24 (68.6)	ns
Age at sampling (year), (median, IQR)	33 (28-47)	36 (26-50)	32 (29-44)	ns
CSF parameters				
Link index	0.6 ± 0.2	0.3± 0.1	0.9 ± 02	<.05
**Oligoclonal bands**				
***Absence***	41 (58.6)	35 (100)	6 (17.1)	<.05
***Presence***	29 (41.4)	0	29 (82.9)	<.05
No. of oligoclonal bands (median, IQR)		/	4 (2-6)	/
Clinical and radiological characteristics				
Disease duration, months (mean ± SD)	/	/	4.2±1.6	
Relapses in the year before diagnosis (mean ± SD)	/	/	1.6±0.5	
Baseline EDSS (median, IQR)	/	**/**	1.0 (1.0-2.0)	
No. of brain MRI lesions on T2 weighted sequences (mean ± SD)	/	**/**	10.5±5.2	
Patients with brain MRI lesions on T1 Gad+ weighted sequences n (%)	/	**/**	22 (62)	
SDMT Z-score (mean ± SD)	/	**/**	0.4±0.2	

* Chi-square tests and Fisher-Exact tests were used to compare categorical variables. Quantitative variables were compared with the Mann–Whitney test.

CSF, cerebrospinal fluid; EDSS, Expanded Disability Status Scale; Gad+ gadolinium; MRI, magnetic resonance imaging; SDMT, symbol digit modalities test; No, number; SD, standard deviation.

### MiRNAs expression levels in CSF

**Figure 1:**
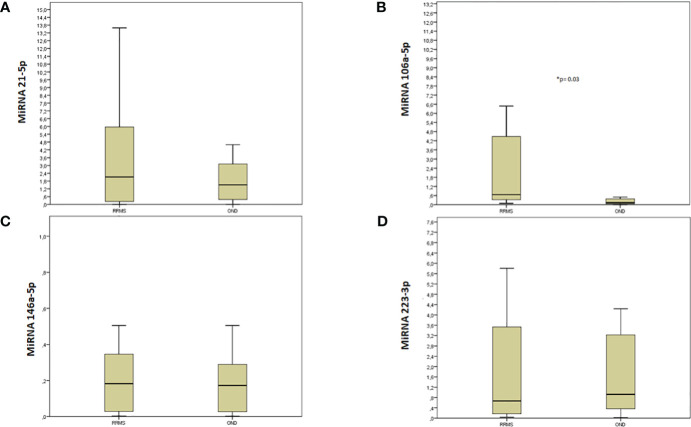
Box plot of MiRNAs expression in RRMS and OND. MiRNAs expression levels in the cell-free CSF of RRMS patients and OND by Real-Time PCR. Quantitative Real-Time PCR analysis of MiRNAs was performed. The relative expression levels were calculated using the comparative Ct method, with miR-16-5p as endogenous control. Data are expressed as mean ± SEM of fold change values. **(A)** miRNA 21-5p; **(B)** miRNA 106a-5p; **(C)** miRNA 146a-5p; **(D)** miRNA 223-3p. *statistically significant.

**Figure 1** reports the box plot comparing the levels of expression of miRNA 21-5p, miRNA 106a-5p, miRNA 146a-5p, and miRNA 223-3p. MiRNA 106a-5p (7.8 ± 7.1 vs 1.3 ± 5.5, p=0.03) had higher levels in RRMS patients when compared to those with OND. The other investigated miRNAs did not show differences in RRMS when compared to OND (miRNA 21-5p 5.9 ± 1.9 vs 4.8 ± 1.5, p= 0.576, miRNA 146a-5p 0.5 ± 0.2 vs 0.4 ± 0.2, p= 0.950, and miRNA 223-3p 3.8 ± 39.1 vs 6.5 ± 19.6, p= 0.148).

**Figure 2:**
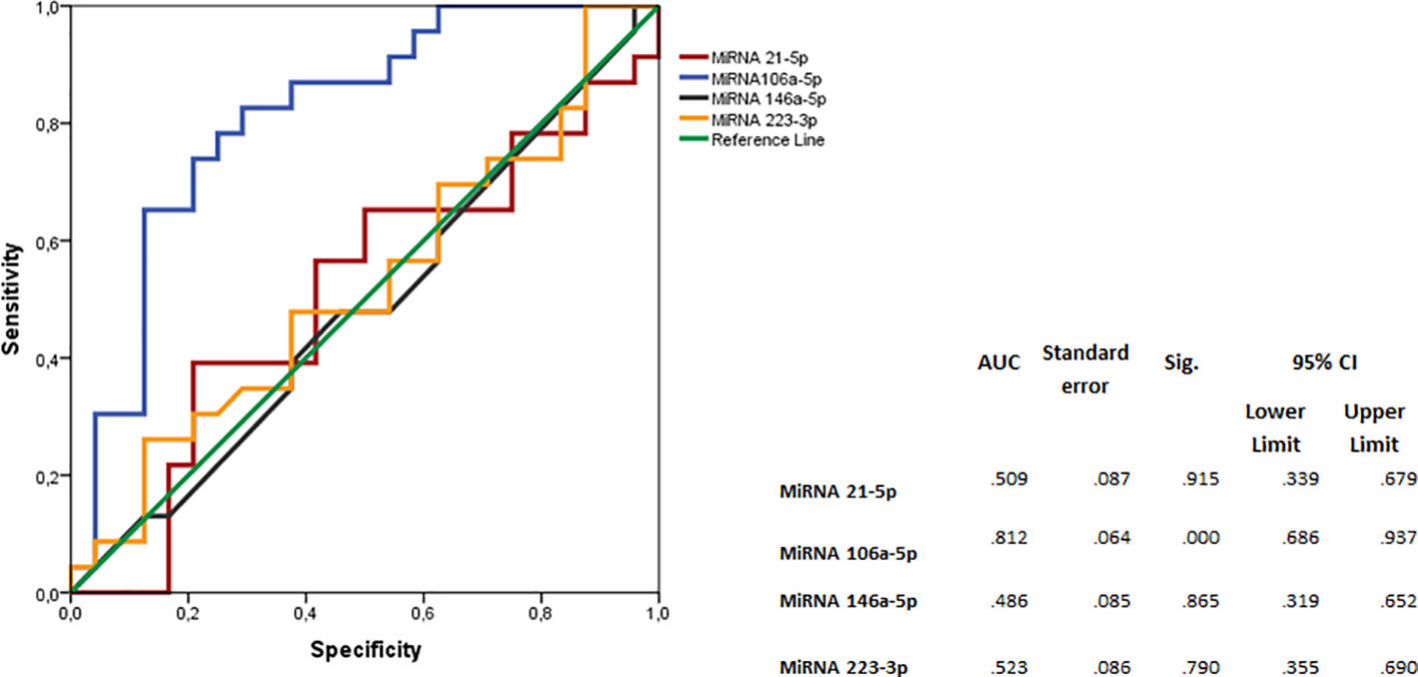
Logistic regression analyses with ROC curve output of patients with RRMS plotted against the OND group. The AUC, with 95% CI, is given for each parameter. The surface expression of each parameter for patients with RRMS (n=35) are combined as true positives and plotted against OND as true negatives (n=35). The diagonal dividing the ROC space represents the random event. A logistic regression analysis with combined parameter results has been performed for “all parameters”, parameters with AUC >0.70, and AUC>0.75. ROC, receiver operating characteristic; AUC, area under the curve; RRMS, relapsing–remitting MS; OND, other neurological diseases.

The ROC curve indicated that MiRNA 106a-5p could be considered as a disease biomarker: AUC 0.812 (p<.001; 95% CI 0.686-0.937) (**Figure 2**).

### Cross-sectional analyses in RRMS patients

Linear regression analysis between MiRNA 106a-5p expression and CSF characteristics showed an association between the number of oligoclonal bands and MiRNA 106a-5p levels (B-coeff 2.6, p<.001; 95% CI 1.3-4.9). Clinical and radiological characteristics and SDMT z scores were not associated with MiRNA 106a-5p expression levels (**Table 2**).

**Table 2:** Linear regression univariable analysis for miRNA106a-5p.

Variables	MiRNA106a-5p β (95% CI)
**Age**	-0.1 (-0.1-0.2), p=0.908
**Disease duration**	-0.2 (-0.1-0.2), p=0.728
**Number of relapses in the year before diagnosis**	0.1 (-4.5-4.8), p=0.944
**EDSS score**	-10.5 (-71.4-50.3), p=0.722
Presence of brain MRI lesions on T1 Gad+ weighted sequences*	-0.4 (-0.2-0.3), p=0.698
**SDMTz score**	-0.4 (-0.8-0.8), p=0.918
**CSF Link Index**	16.2 (-29.1-61.6), p=0.460
**Number of oligoclonal bands**	2.6 (1.3-4.9), p=.027**

CSF, cerebrospinal fluid; EDSS, Expanded Disability Status Scale; Gad+ gadolinium; MRI, magnetic resonance imaging; SDMT, symbol digit modalities test.

*the last category was used as reference; ** statistically significant.

## Discussion

In our cross-sectional real-world study, we found that CSF miRNA106a-5p was upregulated in RRMS patients at the time of diagnosis and that it was associated with a higher number of OCBs.

The role of miRNA 106a-5p in the CSF and blood serum of MS patients has been previously investigated and usually described as downregulated.

Quintana et al. showed a moderate downregulation of the aforementioned miRNA in CSF of MS patients (n=86) compared to those with OND (n=55) (24). In this study, all patients had a recent diagnosis of MS (within one year) but the rate of gadolinium lesion-positive patients (57%) was lower than in our cohort (24). Furthermore, the onset was predominantly monosymptomatic and no data were available on the number of relapses in the year before diagnosis.

An Iranian case-control study examined the hsamiR-106a-5p expression in blood samples of new MS cases or untreated patients (n=32), and they found that it was downregulated in the blood (log fold change = − 1.15/p < 0/05) (8). However, not all the patients were naïve to treatment, no information was provided on the clinical characteristics, and miRNA 106a-5p expression was not examined in cell-free CSF (8).

The gene target interaction network of hsa-miR-106a-5p has been investigated in several settings. In general, miR-106a is induced by TNFα in an NFκb- dependent manner and it is inversely related to TGFβ levels (8, 25, 26). Previous data reports that the downregulation of miR-106a increases inflammatory cytokines, including TNF-α, IL-1β, and IL-628 (8, 26, 27). The study suggested that this increasing level may be due to the MS-related downregulating of hsa-miR-106a-5p (8).

Our results, although opposite to current knowledge on MS cohorts, has been conducted on a homogeneous group of RRMS patients with an early and highly active disease course.

This was also confirmed by the association found with the number of OCBs, conventionally considered as a marker of inflammation (28, 29). In a previous report, MS patients with ≥10 OCBs had significantly higher annualized clinical and radiographic relapse rates (30). However, data about number of OCBs and disease prognosis are controversial (31, 32). On the other hand, another study hypothesized that OCBs (immunoglobulin M) may unveil occult inflammatory processes not detected by conventional MRI studies and that they may be associated to a poorer prognosis (33). Furthermore, in a previous exploratory report, the presence of OCBs at the time of MS diagnosis was associated to a lower retinal thickness (34).

The altered miRNAs expression in early MS phases leads to the modulation of inflammation.

The major strength of this study is to suggest CSF MiRNAs as useful disease biomarkers because of their easy extraction and stability. The characterization of the differential expression of specific miRNA sets could help to provide an early diagnosis, as these molecular factors are more accurate than others.

Future biomarkers may help better and quicker identification and stratification of groups of patients with a highly active or aggressive MS course to personalize therapeutic decisions from the early phases of the disease.

The major limitation of the study is that miRNAs were selected according to previous literature reports without a preliminary phase including complete miRNAs sequencing.

Additionally, the small sample size could have reduced the power of analysis and, furthermore, we did not provide longitudinal clinical data, but only cross-sectional data.

Additional cohort analysis with long term follow-up data is needed to provide further information regarding investigated miRNAs as reliable diagnostic and prognostic biomarkers in MS.

## Data availability statement

The raw data supporting the conclusions of this article will be made available by the authors, without undue reservation.

## Ethics statement

The studies involving human participants were reviewed and approved by Comitato Etico Foggia. The patients/participants provided their written informed consent to participate in this study.

## Author contributions

AZ, VM, GS, ED’A, and CA wrote the manuscript and all authors contributed to the revisions until final draft. AZ and ED’A carried out the detailed planning of the study, supervised by CA. VM was responsible for all laboratory analyses and AZ conducted the statistical analyses. All authors contributed to the article and approved the submitted version.
