# Supplementary material for: MiRNA 106a-5p in cerebrospinal fluid as signature of early relapsing remitting multiple sclerosis: a cross sectional study
Source: Front Immunol. 2023 Aug 30;14:1226130. doi: 10.3389/fimmu.2023.1226130 (PMC10499168; doi:10.3389/fimmu.2023.1226130)
Supplement: Supplementary file 1 [file Table_1.docx]

**Table e1**

**Other neurological diseases (OND) distribution**

| **OND (n=35)** |
| --- |
| **Migraine,** n= 22 (37.1%) |
| **Aspecific Sensory Disturbances**, n=13 (62.9%) |
